# Proteomics Reveals Novel Oxidative and Glycolytic Mechanisms in Type 1 Diabetic Patients' Skin Which Are Normalized by Kidney-Pancreas Transplantation

**DOI:** 10.1371/journal.pone.0009923

**Published:** 2010-03-29

**Authors:** Franco Folli, Valeria Guzzi, Lucia Perego, Dawn K. Coletta, Giovanna Finzi, Claudia Placidi, Stefano La Rosa, Carlo Capella, Carlo Socci, Davide Lauro, Devjit Tripathy, Christopher Jenkinson, Rita Paroni, Elena Orsenigo, Giuliana Cighetti, Luisa Gregorini, Carlo Staudacher, Antonio Secchi, Angela Bachi, Michael Brownlee, Paolo Fiorina

**Affiliations:** 1 Diabetes Division, Department of Medicine, University of Texas Health Science Center at San Antonio, San Antonio, Texas, United States of America; 2 Department of Medicine, Surgery, Dental Science, Università degli Studi di Milano, Milan, Italy; 3 Center for Metabolic Biology, School of Life Sciences, Arizona State University, Tempe, Arizona, United States of America; 4 Department of Pathology, Ospedale di Circolo and Department of Human Morphology, University of Insubria, Varese, Italy; 5 Surgery Department, San Raffaele Scientific Institute, Milan, Italy; 6 Department of Clinical Sciences “Luigi Sacco”, Università degli Studi di Milano, Milan, Italy; 7 Department of Medicine, San Raffaele Scientific Institute, Milan, Italy; 8 Department of Oncology, Università Vita e Salute-San Raffaele, Milan, Italy; 9 Departments of Medicine and Pathology, Albert Einstein College of Medicine, New York, New York, United States of America; 10 Transplantation Research Center/Nephrology Division, Children's Hospital/Brigham and Women's Hospital, Harvard Medical School, Boston, Massachusetts, United States of America; University of Tor Vergata, Italy

## Abstract

**Background:**

In type 1 diabetes (T1D) vascular complications such as accelerated atherosclerosis and diffused macro-/microangiopathy are linked to chronic hyperglycemia with a mechanism that is not yet well understood. End-stage renal disease (ESRD) worsens most diabetic complications, particularly, the risk of morbidity and mortality from cardiovascular disease is increased several fold.

**Methods and Findings:**

We evaluated protein regulation and expression in skin biopsies obtained from T1D patients with and without ESRD, to identify pathways of persistent cellular changes linked to diabetic vascular disease. We therefore examined pathways that may be normalized by restoration of normoglycemia with kidney-pancreas (KP) transplantation. Using proteomic and ultrastructural approaches, multiple alterations in the expression of proteins involved in oxidative stress (catalase, superoxide dismutase 1, Hsp27, Hsp60, ATP synthase δ chain, and flavin reductase), aerobic and anaerobic glycolysis (ACBP, pyruvate kinase muscle isozyme, and phosphoglycerate kinase 1), and intracellular signaling (stratifin-14-3-3, S100-calcyclin, cathepsin, and PPI rotamase) as well as endothelial vascular abnormalities were identified in T1D and T1D+ESRD patients. These abnormalities were reversed after KP transplant. Increased plasma levels of malondialdehyde were observed in T1D and T1D+ESRD patients, confirming increased oxidative stress which was normalized after KP transplant.

**Conclusions:**

Our data suggests persistent cellular changes of anti-oxidative machinery and of aerobic/anaerobic glycolysis are present in T1D and T1D+ESRD patients, and these abnormalities may play a key role in the pathogenesis of hyperglycemia-related vascular complications. Restoration of normoglycemia and removal of uremia with KP transplant can correct these abnormalities. Some of these identified pathways may become potential therapeutic targets for a new generation of drugs.

## Introduction

In type 1 diabetes mellitus (T1D), chronic hyperglycemia leads to the development of both microvascular and macrovascular complications [1], [2]. The Diabetes Control and Complications Trial (DCCT) determined the effect of improved metabolic control in patients with T1D on the development of diabetic complications, such as diabetic retinopathy, nephropathy, neuropathy, and cardiovascular diseases [3]. In a cohort of patients treated with intensive diabetes management, there was a persistent decrease in the incidence of progression in retinopathy as well as of cardiovascular diseases [3], [4].

Experimental data from animal and cellular models have led to several hypotheses on the mechanisms that may link chronic hyperglycemia and diabetic complications, but these hypotheses still have a level of uncertainty with regards to their applicability and significance in explaining complications in patients affected by T1D [2]. The four main mechanisms that may explain how chronic hyperglycemia may induce diabetic complications are: 1) an increase in polyol pathway flux; 2) an increase in advanced glycation end-product formation; 3) an activation of protein kinase C isoforms; and 4) an increase in hexosamine pathway flux [2], [3], [5]. It has been suggested that each of these pathogenic mechanisms may reflect a single hyperglycemia-induced process, specifically, the overproduction of superoxide by the mitochondrial electron-transport chain [5], [6]. It has also been suggested that oxidative stress, which may induce protein modifications altering their activity or function, may accelerate the basic pathogenic processes of diabetic complications [5], [6], [7]. Several major forms of oxidative modifications can occur on amino acid residue side chains, including carbonylation, nitrosylation, and oxidation of methionine to methionine sulfoxide [8]. However, supportive evidence from large clinical trials that shows antioxidants can ameliorate diabetic complications is lacking [3].

Pancreas transplantation is the only treatment in T1D that can restore long-term insulin independence and normoglycemia [9], [10]. In most cases, pancreas transplantation is associated with kidney transplantation in T1D with end-stage renal disease (ESRD). Although these patients need lifelong immunosuppression, successful kidney-pancreas (KP) transplantation can achieve insulin independence as well as a dialysis-free state [9], [10], [11], [12]. The survival rate is almost doubled in KP transplant patients compared with T1D+ESRD patients on the waiting list for a transplant [10], [13]. Interestingly, ultrastructural features of endothelial damage in skin biopsy specimens showed improved profiles in patients who received a successful KP transplant [14].

Proteomics is the combination of two-dimensional polyacrylamide gel electrophoresis (2D PAGE) for protein separation and visualization, followed by mass spectrometric (MS) protein identification using peptide mass fingerprints and tandem MS peptide sequencing [15]. Differential protein expression profiles detected by 2D PAGE and MS have been reported for various types of human diseases and have offered opportunities in identifying new markers and therapeutic targets and new insights in understanding disease pathogenesis [16], [17]. In recent years, the number of novel proteins identified by genomic and proteomic research projects has dramatically increased, with a concomitant more rapid characterization of molecular processes of living cells through large-scale studies in specific biological contexts. We reasoned that the proteomic approach might offer a powerful tool to assess differences in protein expression associated with diabetes and renal complications, as few papers have described the use of this technique in diabetes [18], [19].

Our aim was to study the regulation of protein expression in skin biopsies of patients with T1D and T1D+ESRD, to identify pathways of persistent cellular changes and damage, and to evaluate the effects of restoration of normoglycemia obtained with kidney-pancreas transplantation on these pathways. To this end, we performed two-dimensional electrophoresis and mass spectrometry comparing skin biopsy extracts from the following four groups of patients: controls, T1D, T1D+ESRD, and KP transplanted patients. Proteomic results were integrated with morphological, immunohistochemical, and ultrastructural features of skin tissues of all the patients included in the study. This approach will lead to identification of pathways that can potentially become targets for new class of drugs to control cellular changes associated with vascular abnormalities.

## Materials and Methods

### Ethics Statement

All subjects gave their written informed consent to the study, which was approved by the Ethics Committee of San Raffaele Scientific Institute.

### Patients and Study Design

The study included 11 patients with T1D, 18 with T1D+ESRD, 18 with ESRD who received a simultaneous KP transplant, and 11 healthy subjects (controls).

The study was conducted from June 2000 to June 2004 and all of the transplanted patients that were consecutively admitted to the San Raffaele Hospital in Milan, Italy for the standard check-up were included in the study if they met the inclusion criteria. Only those transplanted patients with a follow-up longer than one year and acceptable graft function were included in the study. Patients with clear signs of systemic infection, lymphoproliferative disease, urinary infection, enhanced erythrocyte sedimentation velocity/C reactive protein were excluded, as well as patients taking oral anticoagulants. Subjects in the four groups were matched for age, gender, diabetes, and dialysis duration (when performed). KP transplant patients were all insulin independent, whereas the T1D and T1D+ESRD patients were on intensive subcutaneous insulin therapy. All of the patients included in the T1D+ESRD and KP groups were on anti-platelet therapy (80% ASA and 20% ticlopidine) to prevent graft or fistula thrombosis. In hemodialyzed patients, blood samples were collected before undergoing dialysis to avoid the confounding effect mediated by heparin administration and by the contact with hemodialysis membrane.

Table 1 displays the laboratory and clinical characteristics of patients. All investigations were performed on skin biopsies obtained from patients by skin-punch biopsy of the internal surface of the arm [20], [21].

**Table 1:** Clinical and laboratory characteristics of the patients enrolled in the study.

	Controls	T1D	T1D+ESRD	KP
Sex (M/F)	7/4	6/5	11/7	11/7
Age (years)	39.4±5.1	37.7±2.7	36.2±2.1	40.1±2.8
HbA1c (%)	4.8±0.1	7.0±0.5*	8.3±0.5*	5.1±0.2
C-peptide (ng/ml)	1.2±0.1	0.1±0.1*	0.1±0.1*	3.0±0.4*
Creatinine (mg/dl)	1.1±0.2	0.9±0.2	8.3±0.4*	1.4±0.1
Insulin (µ IU/ml)	5.1±2.5	10.3±1.9*	26.9±7.1*	14.9±1.4*
BMI (Kg/m^2^)	25.1±0.5	23.1±1.0	22.6±0.5	24.6±0.6
HOMA index	2.3±0.1	2.9±0.4	6.4±1.6*	3.1±0.3*
T1D duration (years)	/	25.4±1.1	27.3±1.4	24.5±1.2
Transplant duration (years)	/	/	/	5.6±1.1
Retinopathy	0/11	11/11	17/18	18/18
Nephropathy	0/11	3/11	18/18	18/18
Neuropathy	0/11	11/11	18/18	18/18
Cardio-cerebro-vascular diseases	0/11	0/11	2/18	3/18

*: statistical significance (p<0.05) versus control; T1D (type 1 diabetic patients); T1D+ESRD (T1D+end-stage renal disease); KP (kidney-pancreas transplanted patients); HbA1c (glycated hemoglobin); BMI (body mass index); HOMA (homeostatic model assessment).

### Transplantation and Immunosuppression

Organs for transplantation were obtained from deceased donors through the “North Italia Transplant” organ procurement consortium (NITp, Milan, Italy). After induction with ATG (thymoglobulin, IMTIX, SANGSTAT), immunosuppression was maintained using cyclosporine (through levels between 100–250 ng/ml) or FK506 (through levels between 10–15 ng/ml), mycophenolate mofetil (500–2000 mg/day), and methylprednisolone (10 mg/day). Steroids were withdrawn within 3–6 months after transplantation. Episodes of kidney rejection were treated with pulses of 500 mg of methylprednisolone. Cases of “steroid-resistant” rejection were treated with OKT3 or a course of ATG.

### 2D-Electrophoresis

Tissues were homogenized in a lysis buffer consisting of 10 mM Hepes, 1% Triton X-100, and protease inhibitor cocktail, including aprotinin, leupeptin, pepstatin A, Bestatin, E-64, AEBSF, PMSF, or benzamidine (Sigma) (10 µl/ml). Proteins were extracted at 4°C, protein concentration was determined using the Bio-Rad protein assay, and pools of two biopsies were made for each experiment. For the 2D-polyacrylamide gels, 250 µg of extract were precipitated with four volumes of acetone and resuspended in a running buffer consisting of 8 M urea, 4% CHAPS, 65 mM DTT, BFB 0.05% (w/v), and IPG buffer 1.7% (v/v). Samples of 100 µg were analyzed by 2D IEF/SDS–PAGE Immobiline IPG strips DryStrips (18 cm 3–10 pH non-linear, Amersham Biosciences) on an IPGphor apparatus according to the manufacturer's instructions (Amersham Biosciences). After rehydration (1 hour at 18°C) of the IPG strip and absorption of the sample (30 V, for 8 hours at 18°C), isoelectric focusing was performed using a voltage gradient from 300 to 3,500 V for three hours, followed by 3,500 V for another three hours (according to the manufacturer's instructions). For the second dimension, the IPG strip was equilibrated for 15 minutes in equilibration buffer containing DTT 2% w/v and then for 15 minutes in equilibration buffer containing iodoacetamide 2.5% w/v (equilibration buffer consisted of urea 6 M, glycerol 30% v/v, SDS 2% w/v, and 50 mM Tris/HCl pH 8.8). Protein separation in the second dimension was performed in gradient (9%–16.5%) polyacrylamide gels. Theses gels were fixed twice in two solutions containing different concentrations of methanol and acetic acid (40% methanol/10% acetic acid; 5% methanol/5% acetic acid) and then washed with deionized water (3×20 minutes). The sensitization step was performed with 0.02% sodium thiosolphate. After two washing steps (1 minute ×2), the gels were stained with silver-staining solution without glutaraldehyde (0.1% silver nitrate) and then washed for one minute with deionized water. The gels were developed in a solution containing 2.5% Na_2_CO_3_, 0.008% formaldehyde. Reaction was stopped with 5% acetic acid [22]. An Image Scanner was used to scan the gels and then they were analyzed with Image Master 2D Elite software (Amersham Biosciences). Protein expression in diabetic and transplanted patients was normalized with healthy controls. Protein expression in diabetic and transplanted patients was normalized with healthy controls as 100% in the case of down-regulated proteins, or with diabetic and diabetic-uremic patients at 100% in the case of proteins that were up-regulated in these patients' categories. Nine independent experiments were performed with pools of proteins from various skin donors.

### Protein Identification by MALDI-TOF MS Analysis

Spots of interest were excised from silver-stained gels either by manual or automated excision (ProXCISION; PerkinElmer), reduced, alkylated, and digested overnight with bovine trypsin (Roche), as previously described [23], [24]. One micro-liter aliquots of the supernatant were used for MALDI-TOF MS analysis (Voyager-DE STR from Applied Biosystem, Framingham, MA), using the dried droplet technique and cyano-4-hydroxycinnamic acid as matrix. Alternatively, gel fragments were further extracted and the resulting peptide mixture was subjected to a single desalting/concentration step before the mass spectrometric analysis over μZipTipC_18_ (Millipore Corporation, Bedford, MA). MALDI-TOF spectra were internally calibrated using trypsin autolysis products and processed via Data Explorer software. Proteins were unambiguously identified by searching in the comprehensive non-redundant protein database with the program ProFound [16], [17]. One missed cleavage per peptide was allowed, and an initial mass tolerance of 50 ppm was used in all searches.

### Statistical Analysis

Data were analyzed using SPSS statistical package for Windows, 10.1 (SPSS Inc., Chicago, IL). Quantitative data were expressed as mean ± standard error and were tested for normal distribution with the Kolmogorov-Smirnov test and for homogeneity of variances with Levene's test. When more than two groups were compared cross-sectionally, ANOVA (for parametric data) or Kruskal-Wallis test (for non-parametric data) was used according to distribution. When ANOVA was used, multiple post-hoc comparison analysis was performed with Tukey's test. A *P* value of less than 0.05 (by two-tailed testing) was considered of statistical significance. Further information concerning the materials/instruments utilized, antibodies used to characterize skin biopsy specimens, pathway analysis, immunohistochemistry, electron microscopy, and the malondialdehyde and GSH quantification can be found in the Supporting Information [File S1].

## Results

### Proteomic analysis revealed alterations in three major groups of proteins in T1D and T1D+ESRD patients

**Figure 1:**
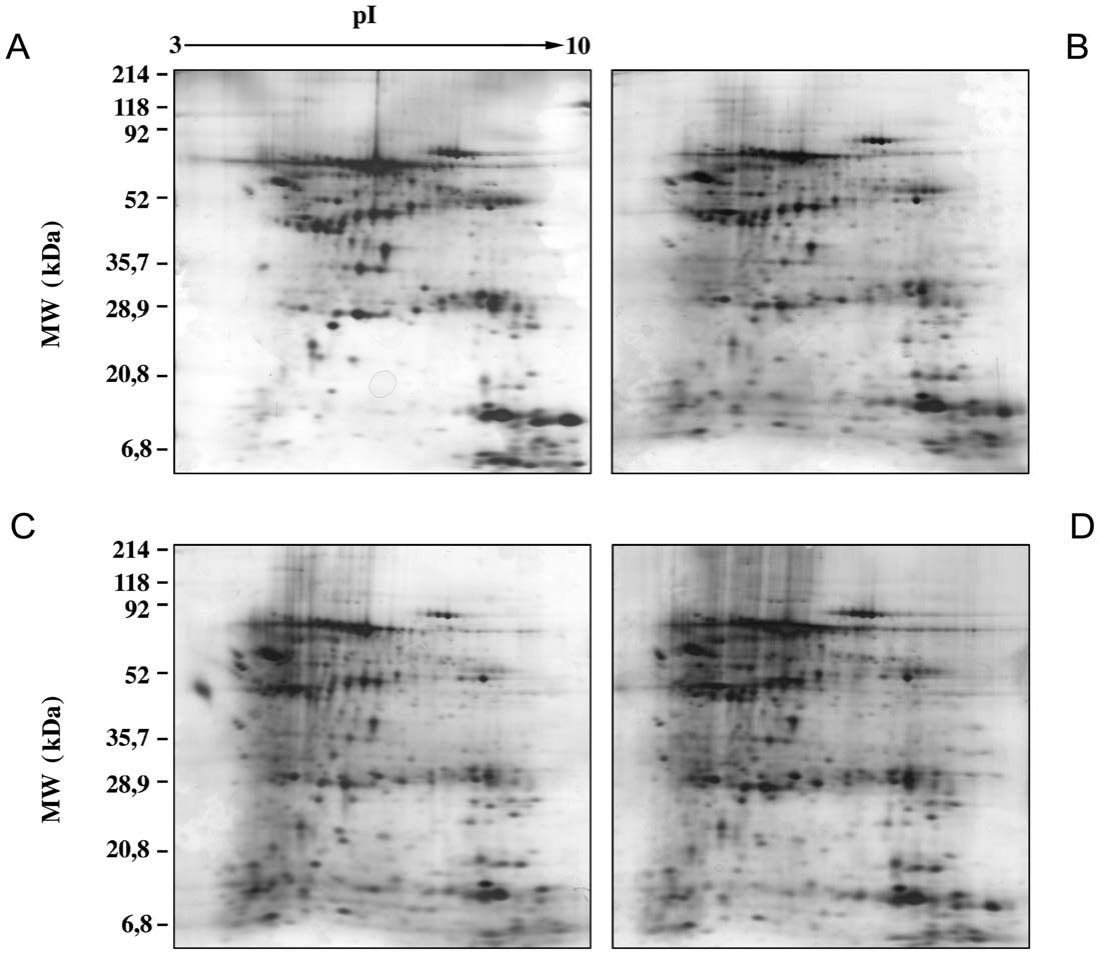
Typical silver-stained 2D electrophoresis pattern of proteins isolated from human skin biopsies in the broad nonlinear pH range 3–10. Skin biopsies were obtained from controls, patients affected by type 1 diabetes (T1D), patients affected by T1D and end-stage renal disease (T1D+ESRD), and kidney-pancreas transplanted patients (KP).

**Figure 2:**
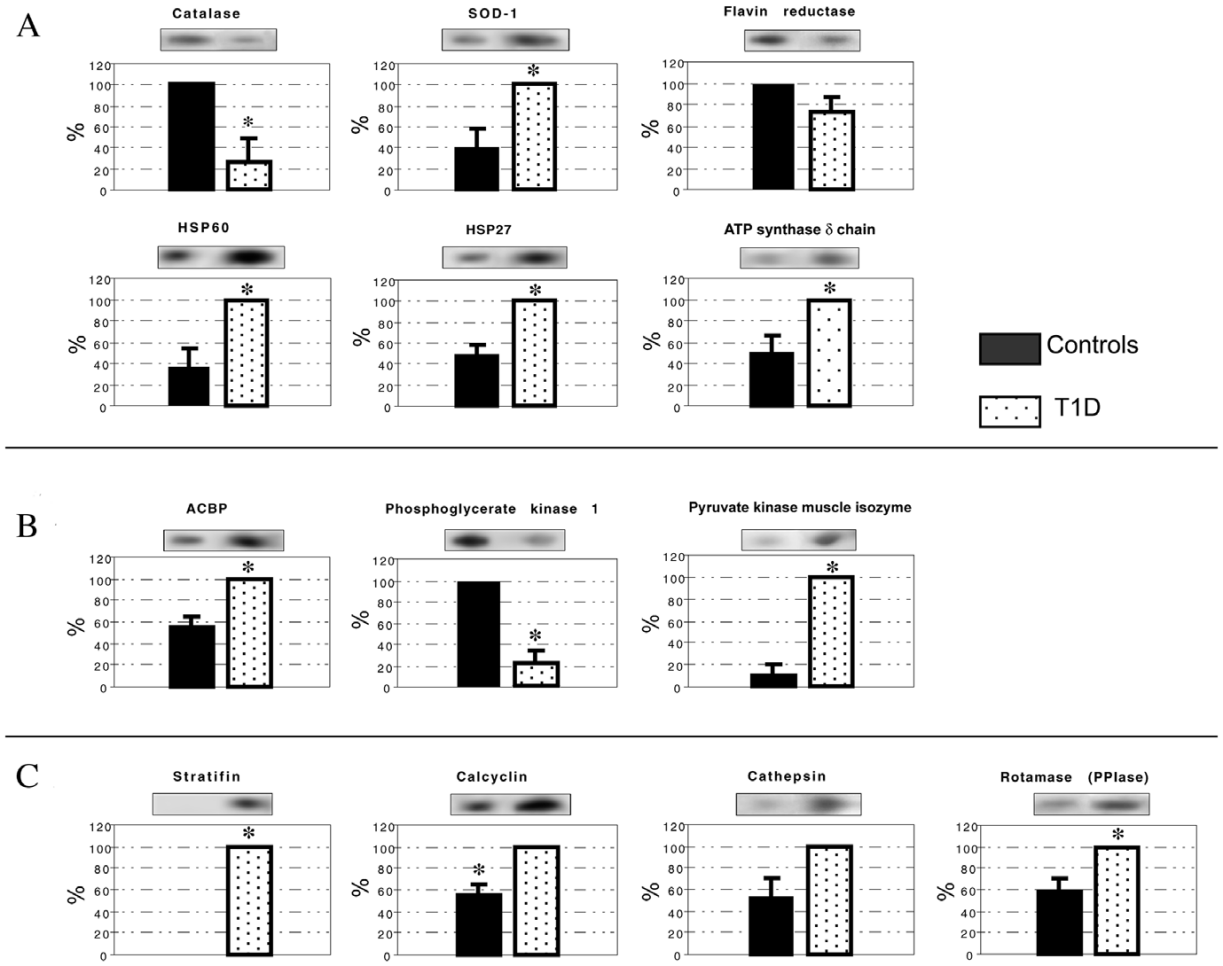
Densitometric quantitation of differential protein expression in controls and type 1 diabetic patients (T1D). Representative protein spots from 2D gels are inserted above each bar chart. SOD1 expression was increased by 2.5-fold and ATP synthase δ chain 2-fold in T1D compared with controls (p<0.01) (A). HSP27 and HSP60 were both up-regulated in T1D patients compared with controls (p<0.01) (A). Pyruvate kinase (10-fold) (p<0.001) and ACBP (2-fold) (p<0.01) expression in T1D patients was up-regulated compared with controls (B); while stratifin was not detectable in control patients and highly expressed in both T1D groups (p<0.001) (C). S100-calcyclin and rotamase expression was up-regulated by 2-fold in T1D compared with controls (p<0.05) (C). However, T1D showed a decrease in phosphoglycerate kinase and catalase expression compared with controls (p<0.05) (A, B). Flavin and Cathepsin are unchanged (A, C).

**Figure 3:**
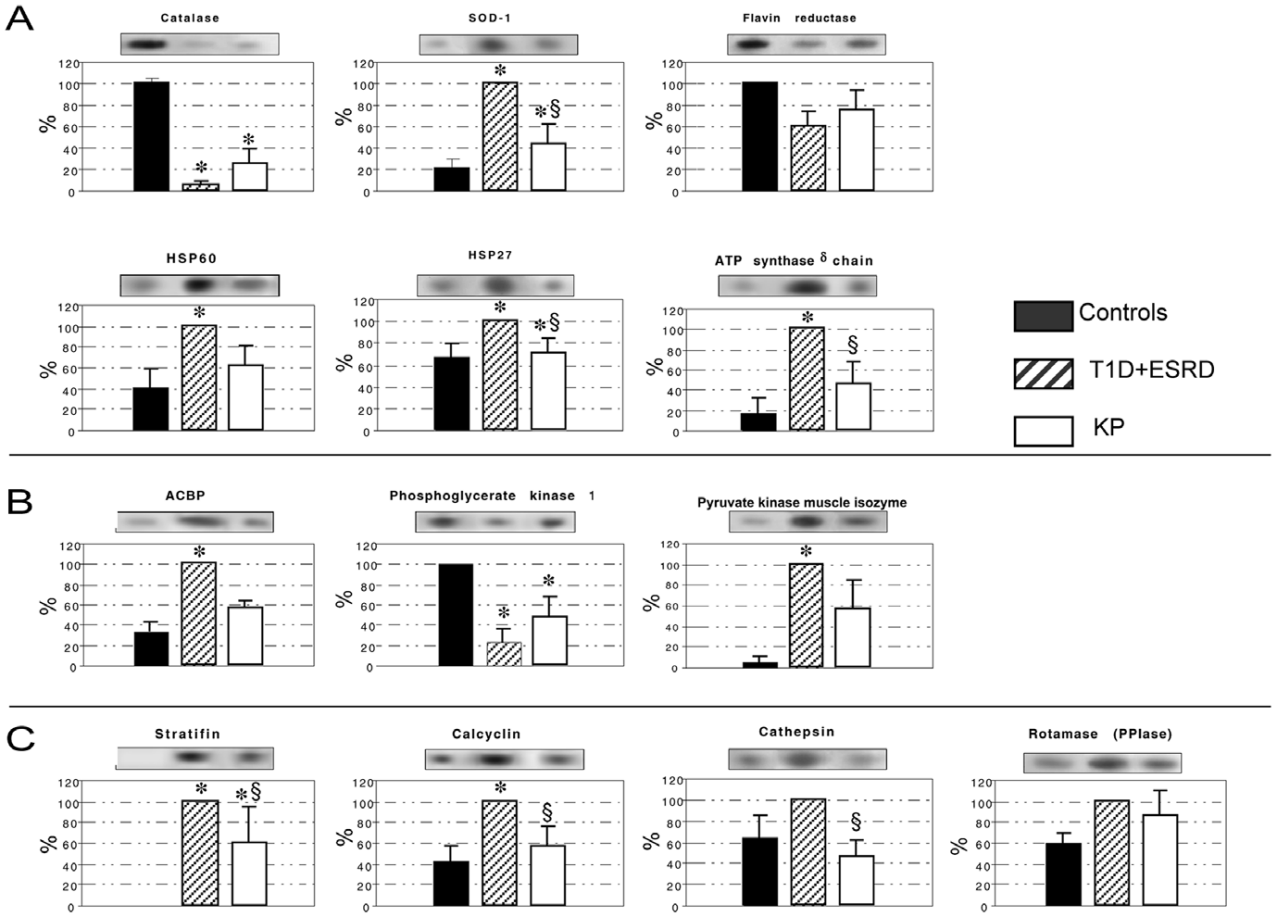
Densitometric quantitation of differential protein expression in controls, type 1 diabetic patients with end-stage renal disease (T1D+ESRD), and kidney-pancreas transplanted patients (KP). Representative protein spots from 2D gels are inserted above each bar chart. SOD1 expression was increased by 5-fold in T1D+ESRD patients compared with controls (p<0.01). KP transplantation was shown to reduce by 2-fold this up-regulation (KP vs. T1D+ESRD and vs. controls, p<0.01 and p<0.05, respectively) (A). HSP27 expression was increased in T1D+ESRD patients compared with controls (p<0.01), and it was almost normalized in the KP group (KP vs. T1D+ESRD p<0.05). HSP60 expression was increased more than 2-fold in T1D+ESRD group compared with controls (p<0.01) and normalized in KP patients (KP vs. T1D+ESRD p<0.01) (A). ATP-synthase δ chain was 6-fold up-regulated in T1D+ESRD patients compared with controls (p<0.001) and was partially normalized in KP patients (KP vs. controls and T1D+ESRD p<0.05 and p<0.01) (A). The expression of pyruvate kinase (B) and stratifin (C) was increased in T1D+ESRD patients compared with controls (p<0.001). KP transplantation reduced the expression of pyruvate kinase (KP vs. T1D+ESRD p<0.05) and stratifin (KP vs. controls p<0.01 and vs. T1D+ESRD p<0.05) compared with T1D+ESRD. ACBP expression was increased by 3-fold in T1D+ESRD patients compared with controls (p<0.01) while in KP group the levels normalized (B). S100-calcyclin expression was up-regulated by 2-fold (p<0.01 vs. controls), while rotamase and cathepsin were unchanged (C). S100-calcyclin expression was normalized in KP group (p<0.05) (C). Catalase expression decreased 25-fold (p<0.001) (A) and phosphoglycerate kinase 1 expression decreased 4-fold (p<0.01) (A) in T1D+ESRD group compared with controls. A clear reversal of phosphoglycerate kinase 1 and partially of catalase expression abnormalities was observed after KP transplantation (p<0.05 and p<0.01 vs. T1D+ESRD for phosphoglycerate kinase 1 and partially of catalase, respectively), (B). Flavin reductase expression was halved in T1D+ESRD compared with controls (p<0.05), (A).

We extracted proteins from skin biopsies of the four groups and performed two-dimensional electrophoresis comparing skin biopsy extracts of healthy control subjects, T1D, T1D+ESRD, and KP transplant patients. Figure 1 shows a typical silver-stained 2D electrophoresis pattern of proteins isolated from human skin biopsies in the broad non-linear pH range 3–10. In each gel approximately 200 silver-stained spots were detected, matched, and quantified using image analysis software (Image Master software, Amersham Biosciences). We evaluated the total number of spots in the gels observing high reproducibility of all the experiments performed and high similarity of the gels in each experiment. The results were processed and the expression of albumin and keratin was quantified as a positive control. These proteins presented comparable expression in the four groups of patients (data not shown). However, the analysis with Image Master 2D Elite software emphasized that there were some spots differentially expressed in T1D+ESRD and T1D compared with the controls. These spots were subjected to tryptic digestion and mass spectrometric analysis in order to identify the proteins. MALDI-TOF-MS analysis revealed that 19.5% was albumin, 5.4% hemoglobin, 3.3% keratin, and 6.5% anti-trypsin precursor protein. The remaining 65.3% are listed in Table 2. A number of proteins were not identified because of insufficient material for the mass analysis and/or incomplete correspondence in the protein databases. Only three groups of proteins were significantly aberrantly expressed in T1D and T1D+ESRD: (i) Protein linked to oxidative stress response (catalase, superoxide dismutase 1/SOD-1, flavin reductase, HSP60, HSP27, and ATP synthase δ chain) (Figures 2A and 3A); (ii) protein linked to aerobic and anaerobic glycolysis (ACBP, phosphoglycerate kinase 1 and pyruvate kinase muscle isozyme) (Figures 2B and 3B); and (iii) protein related to intracellular signaling pathways (stratifin-14-3-3, S100-calcyclin, cathepsin, and PPI rotamase), (Figures 2C and 3C).

**Table 2:** Proteins identified by MALDI-TOF-MS analysis.

SwissProt accession no.	PROTEIN	Measured peptides	Matched peptides	Seq. cov. %
P02768	albumin	13	11	22
P01922	hemoglobin/Hemoglobin alpha chain	7	4	35
P13645	keratin	16	7	11
P01009	anti-trypsin precursor protein	27	13	25
P07108	Acyl-coa-binding protein (ACBP)	14	5	47
P04217	Alpha 1b-glycoprotein precursor	11	8	26
P02652	Apolipoprotein A-II precursor	13	4	27
P06576	ATP synthase δ chain	59	28	47
O75947	ATP synthase δ chain	11	6	34
P06703	Calcyclin; S100 calcium-binding protein A6	20	11	51
Q9NZT1	Calmodulin-like skin protein	7	6	42
P04040	Catalase	14	13	39
P07339	Cathepsin D	10	7	15
P02787	Serotransferrin	9	6	9
P06733	Enolase 1 (Phosphopyruvate hydratase)	23	22	52
P10768	Esterase d	9	6	34
P04792	Heat shock protein 27 kda	14	10	48
P10809	Heat shock protein 60 kda	47	32	54
P30043	NADPH-flavin reductase	16	11	67
P05092	Peptidyl-prolyl cis-trans isomerase A (Rotamase)	15	9	39
P32119	Peroxiredoxin 2	9	8	40
P30044	Peroxiredoxin-5, mitochondrial;	27	7	50
P06733	2-phosphopyruvate-hydratase alpha-enolase	5	5	15
P00558	Phosphoglycerate kinase 1	7	5	21
P02647	Proapolipoprotein	39	30	80
P14618	Pyruvate kinase muscle isozyme	14	8	14
P31947	Stratifin	17	9	29
P00441	Superoxide dismutase-1	9	5	40
P02766	Transthyretin precursor	8	6	50
P02766	Transthyretin precursor	8	5	53
P00938	Triosephosphate isomerase 1	33	15	69

### Protein linked to oxidative stress response and aerobic/anaerobic glycolysis are up-regulated in T1D and T1D+ESRD patients compared with controls

#### (i) T1D vs. controls

We first evaluated the effect of long-standing T1D on protein expression in the absence of ESRD, which alone, can enhance oxidative stress. Skin biopsies obtained from T1D patients with normal kidney function were subjected to proteomic analysis. SOD-1 expression was increased 2.5-fold and ATP synthase δ chain 2-fold in T1D patients compared with controls (p<0.01), (Figure 2A). HSP60 and HSP27 were both up-regulated in T1D patients compared with controls (p<0.01), (Figure 2A). Pyruvate kinase (10-fold) (p<0.001) and ACBP (2-fold) (p<0.01) expression in T1D patients was up-regulated compared with controls (Figure 2B); while stratifin was not detectable in control patients it was highly expressed in T1D (p<0.001), (Figure 2C). S100-calcyclin and rotamase expression was up-regulated by 2-fold in T1D patients compared with controls (p<0.05), (Figure 2C). On the other hand, T1D patients showed a decrease in phosphoglycerate kinase and catalase expression compared with controls (p<0.05), (Figures 2A and 2B). Cathepsin and flavin were unchanged (Figures 2A and 2C).

#### (ii) T1D+ESRD patients compared with controls and KP transplant patients

We then evaluated the effect of T1D and ESRD on protein expression and the effect of KP transplantation with restoration of normoglycemia and the withdrawal of uremia. SOD1 expression was increased by 5-fold in T1D+ESRD patients compared with controls (p<0.01). KP transplantation led to a reduction in the up-regulation of SOD1 expression by 2-fold (KP vs. T1D+ESRD and vs. controls, p<0.01 and p<0.05, respectively), (Figure 3A). HSP27 expression was increased in T1D+ESRD patients compared with controls (p<0.01) and it was almost normalized in the KP group (KP vs. T1D+ESRD p<0.05). Hsp60 expression was increased more than 2-fold in T1D+ESRD patients compared with controls (p<0.01) and was normalized in KP patients (KP vs. T1D+ESRD p<0.01), (Figure 3A).

ATP-synthase δ chain was 6-fold up-regulated in T1D+ESRD patients compared with controls (p<0.001) and was partially normalized in KP patients (KP vs. controls and T1D+ESRD p<0.05 and p<0.01), (Figure 3A). The expression of pyruvate kinase (Figure 3B) and stratifin (Figure 3C) was increased in T1D+ESRD patients compared with controls (p<0.001). KP transplantation reduced the expression of pyruvate kinase (KP vs. T1D+ESRD p<0.05) and stratifin (KP vs. controls p<0.01 and vs. T1D+ESRD p<0.05) compared with T1D+ESRD patients. ACBP expression was increased by 3-fold in T1D+ESRD patients compared with controls (p<0.01) while in the KP group the levels normalized (Figure 3B). S100-calcyclin expression was up-regulated by 2-fold (p<0.01 vs. controls), while rotamase and cathepsin were unchanged (Figure 3C). S100-calcyclin expression was completely normalized by KP transplant (p<0.05), (Figure 3C). Three proteins were significantly down-regulated in KP patients: catalase, phosphoglycerate kinase 1, and flavin reductase. Catalase expression decreased about 25-fold (p<0.001), (Figure 3A) and phosphoglycerate kinase 1 expression almost 4-fold (p<0.01), (Figure 3B) in T1D+ESRD patients compared with controls. A clear reversal of phosphoglycerate kinase 1 and partially of catalase expression abnormalities was observed after KP transplantation (p<0.05 and p<0.01 vs. T1D+ESRD for phosphoglycerate kinase 1 and partially of catalase, respectively). Flavin reductase expression was reduced by half in T1D+ESRD patients compared with controls (p<0.05), with a near normalization (ns vs. controls, Figure 3A).

By comparing the proteomic pattern in T1D and T1D+ESRD patients, it appeared that protein expression abnormalities are consistently present in both groups at a different degree. As expected, ESRD increased the alterations of HSP and anti-oxidative machinery in T1D.

### Immunohistochemical and ultrastructural analysis

**Figure 4:**
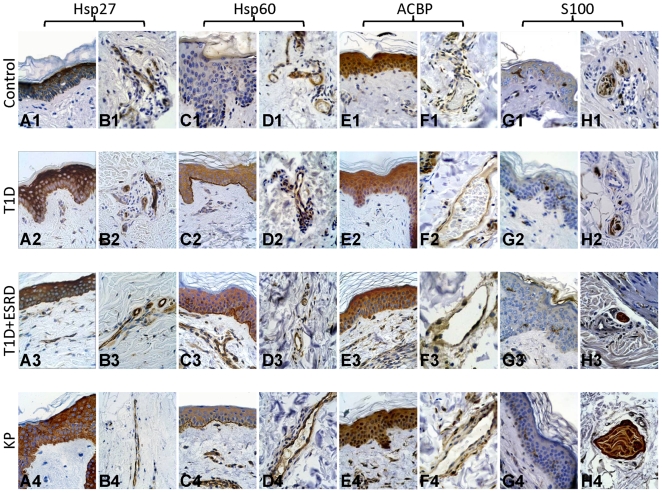
Immunohistochemical expression of Hsp27, Hsp60, ACBP, and S100 proteins in controls, patients affected by type 1 diabetes (T1D), type 1 diabetes+end-stage renal disease (T1D+ESRD), and kidney-pancreas transplanted patients (KP) skin specimens. Hsp27 was strongly expressed in the cytoplasm of all epidermal cells without significant differences among the four categories of patients (A1–A4). Endothelial cells of control, T1D, and T1D+ESRD patients were intensely immunoreactive for Hsp27 (B1–B3), while in KP patients the endothelial Hsp27 immunoreactivity was weaker (B4). Hsp60 immunoreactivity was not found in the epidermal layer of control patients (C1), while it was expressed in the epidermal cells of T1D, T1D+ESRD, and KP patients (C2–C4), although the intensity of the reaction was lower than that of Hsp27. Hsp60 immunoreactivity was also found in endothelial cells of all patient groups, but the immunoreactivity was slightly more intense in control and KP patients (D1, D4) than in T1D and T1D+ESRD patients (D2, D3). ACBP immunoreactivity was intense and diffuse in epidermal epithelial cells of all four patient groups (E1–E4) without significant differences, while it was weaker in endothelial cells of the same patients (F1–F4). S100 protein immunoreactivity was intense in dendritic Langerhans cells distributed among epithelial cells of the epidermal layer (G1–G4) and in nerves (H1–H4) of all patients studied.

Although immunohistochemistry is not a quantitative method able to differentiate protein levels in skin specimens among the four categories studied (control, T1D, T1D+ESRD, KP patients), it allowed us to exactly localize the HSP complex and oxidative stress-related proteins in skin compartments (Figure 4 and Table 3).

**Table 3:** Results of the immunohistochemical analysis performed in the skin biopsies from control, patients affected by type 1 diabetes (T1D), T1D+end-stage renal disease (ESRD), and kidney-pancreas transplanted (KP) patients.

Antibody	Epidermis				Endothelial cells				Muscular cells				Nerves			
	Control	T1D	T1D+ESRD	KP	Control	T1D	T1D+ESRD	KP	Control	T1D	T1D+ESRD	KP	Control	T1D	T1D+ESRD	KP
Hsp27	+++	+++	+++	+++	+++	+++	+++	+	−	+	+	+	−	−	−	−
Hsp60	−	+	+	+	+++	++	+	+++	−	+	−	−	+	+	+	+
ACBP	+++	+++	+++	+++	+	+	+	+	−	−	−	−	−	−	−	−
S100	+*	+*	+*	+*	−	−	−	−	−	−	−	−	+++	+++	+++	+++
Catalase	−	−	−	−	−	−	−	−	+	−	+++	+	−	−	−	−
PRXV	−	−	−	−	−	−	−	−	+++	+++	+++	+++	−	−	−	−
Cathepsin D	+++	+	+++	+++	−	−	−	+	−	−	−	−	−	−	−	−

+++ (intense immunoreactivity); + (weak immunoreactivity); − (no immunoreactivity).

*(dendritic Langerhans cells).

Immunohistochemical expression of Hsp27, Hsp60, ACBP, and S100 proteins was evaluated in control, T1D, T1D+ESRD, and KP skin specimens. Hsp27 was strongly expressed in the cytoplasm of all epidermal cells without significant differences among the four categories of patients (Figures 4A 1–4A4). Endothelial cells of control, T1D, and T1D+ESRD patients were intensely immunoreactive for Hsp27 (Figures 4B 1–4B3), while in KP patients the endothelial Hsp27 immunoreactivity was moderate (Figure 4B 4). Hsp60 immunoreactivity was not found in the epidermal layer of control patients (Figure 4C 1), while it was expressed in the epidermal cells of T1D, T1D+ESRD, and KP patients (Figures 4C 2–4C4), although the intensity of the reaction was lower than that of Hsp27. Hsp60 immunoreactivity was also found in endothelial cells of all patient groups, but the immunoreactivity was slightly more intense in control and KP patients (Figures 4D 1 and 4D4) than in T1D and T1D+ESRD patients (Figures 4D 2 and 4D3). ACBP immunoreactivity was intense and diffuse in epidermal epithelial cells of all four patients groups (Figures 4E 1–4E4) without significant differences, while it was weaker in endothelial cells of the same patients (Figures 4F 1–4F4). S100 protein immunoreactivity was intense in dendritic Langerhans cells distributed among epithelial cells of the epidermal layer (Figures 4G 1–4G4) and in nerves (Figures 4H 1–4H4) of all patients studied.

**Figure 5:**
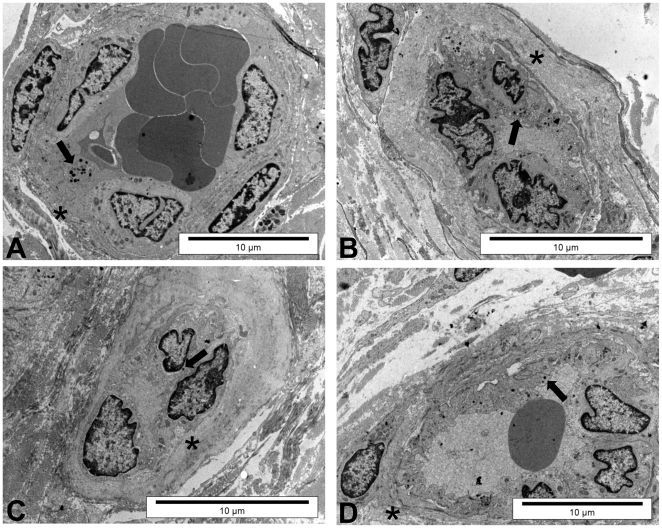
Ultrastructural features of skin tissues. In the control group (A) the vessel lumen is correctly dilated and the endothelial cells are well preserved showing Weibel-Palade granules (arrow). The basal membrane is thin (asterisk). In skin specimens from patients affected by type 1 diabetes (T1D) (B), the vessel lumen is collapsed, endothelial cells show some degenerative markers, such as pre-apoptotic nuclei, dilated reticulum, ramificated microvilli, and rare Weibel-Palade granules (arrow). The thickness of the basal membrane is also remarkable (asterisk). Skin from patients with T1D and end-stage renal disease (T1D+ESRD) (C) showed endothelial cells with numerous signs of damage, including pre-apoptotic nuclei, marked bundles of intermediate filaments, dilated cysternae of reticulum, ramificated microvilli, and very rare Weibel-Palade granules (arrow). In addition, the basal membrane is very thick (asterisk). Skin from kidney-pancreas transplanted patients (KP) (D) showed reversal of almost all injury features: the basal membrane less thick (asterisk), lightly collapsed lumen, rare microvillar ramification, and presence of Weibel-Palade granules (arrow).

The ultrastructural characteristics of T1D, T1D+ESRD, and KP groups compared with controls are represented in Figure 5. The basal membrane of vessels was at least 3-fold thicker in T1D (1986.3±352.1 nm) and T1D+ESRD patients (2185.0±330.4) compared with controls (711.0±172.3 nm) (p = 0.01 and p = 0.02, controls vs. T1D and T1D+ESRD, respectively) (Figures 5A–5D and Table 4). The collapse of the lumen vessel was more evident in the T1D group (2.0±0.4 AU) and T1D+ESRD (2.3±03 AU) compared with controls (0.5±0.2 AU) (p = 0.01 and p = 0.001, controls vs. T1D and T1D+ESRD, respectively) (Figures 5A–5D and Table 4). Microvillar ramification was increased in T1D+EDRD group (2.8±0.1 AU) compared with controls (1.5±0.3 AU) (p = 0.03 controls vs. T1D+ESRD), (Figures 5A–5D and Table 4). Endothelial cells of the T1D showed numerous signs of damage as the presence of bundles of intermediate filaments and pre-apoptotic nucleus (Figure 5B and Table 4). Moreover, the cysternae of endoplasmic reticulum were slightly dilated and the Weibel-Palade granules were increased (Figure 5B and Table 4). Interestingly, KP transplanted patients had a marked improvement of almost all of these ultrastructural cell features which were not statistically different from control subjects (Figure 5D and Table 4). These findings confirm that KP transplantation can modify even long-term established lesions in T1D and T1D+ESRD patients and allow us to generate a close link between these ultrastructural features and the reversal of persistent changes at cellular levels.

**Table 4:** Quantification of ultrastructural features as evaluated on vessels obtained skin biopsies.

	Controls	T1D	T1D+ESRD	KP
Basal membrane thickness (nm)	711.0±172.3	1986.3±352.1*	2185.0±330.4#	1292.0±282.3
Collapsed vessel lumen (AU)	0.5±0.2	2.0±0.4^o^	2.3±0.3$	1.1±0.1
Microvillar ramification (AU)	1.5±0.3	2.3±0.3	2.8±0.1^oo^	1.8±0.4
Reticulum cysternae dilatation (AU)	1.1±0.3	1.6±0.3	1.6±0.4	1.3±0.3
Intermediate filaments (AU)	2.5±0.3	3.0±0.3	2.0±0.3	2.5±0.2
Weibel Palade granules (AU)	2.8±0.1	2.8±0.1	2.3±0.3	2.3±0.3
Pre-apoptotic nuclei (AU)	1.5±0.3	1.6±0.3	2.3±0.2	2.3±0.3

T1D (type 1 diabetic patients).

*p = 0.01 and.

# p = 0.002 compared with controls; T1D and T1D+ESRD (T1D+end-stage renal disease).

o p = 0.01 and.

$ p = 0.001 compared with controls; T1D+ESRD.

oo p = 0.03 compared with controls; KP (kidney-pancreas transplanted patients); AU (arbitrary units).

### Evaluation of oxidative stress

**Figure 6:**
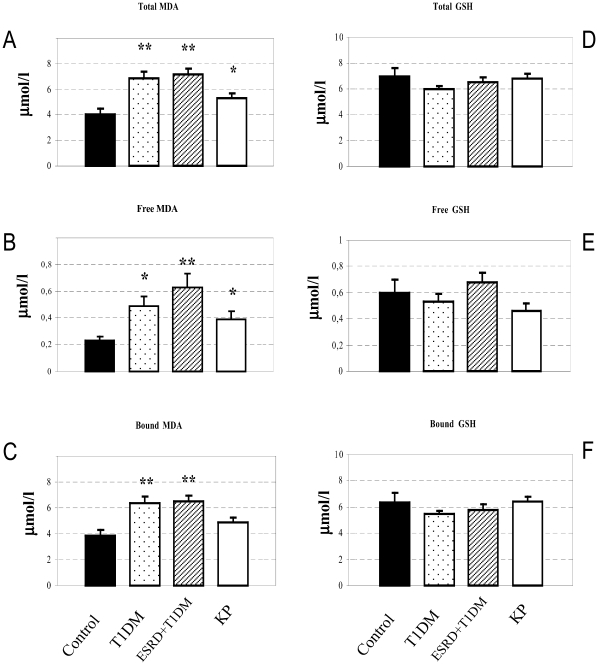
Peripheral levels of total, free and bound malondialdehyde. Patients affected by type 1 diabetes (T1D) and T1D+ end-stage renal disease (T1D+ESRD) demonstrated an increase in total (p<0.01) (A), free (p<0.05 T1D vs. controls and p<0.01 T1D+ESRD vs. controls, respectively) (B), and bound malondialdehyde (MDA) (p<0.01) (C). The levels of total (ns), free (p<0.05), and bound MDA (p<0.05) in kidney-pancreas transplanted patients (KP) was comparable to or slightly increased the control group, indicating a profound effect of kidney-pancreas transplantation in correcting increased oxidative stress (A–C). In contrast, no significant differences were evident in the GSH/GSSG ratio among the four groups (D–F).

Malondialdehyde (MDA), widely used to monitor oxidative stress [25], was evaluated in our four groups. T1D and T1D+ESRD patients demonstrated an increase in total (p<0.01) (Figure 6A), free (p<0.05 T1D vs. controls and p<0.01 T1D+ESRD vs. controls, respectively) (Figure 6B), and bound MDA (p<0.01) (Figure 6C). The levels of total (ns), free (p<0.05), and bound MDA (p<0.05) in KP transplant patients was comparable or slightly increased compared with the control group, indicating a profound effect of KP transplantation in correcting increased oxidative stress (Figures 6A–6C). In contrast, no significant differences were evident in GSH/GSSG (glutathione and glutathione disulfide) ratio among the four groups (Figures 6D–6F).

### Pathway analysis

**Figure 7:**
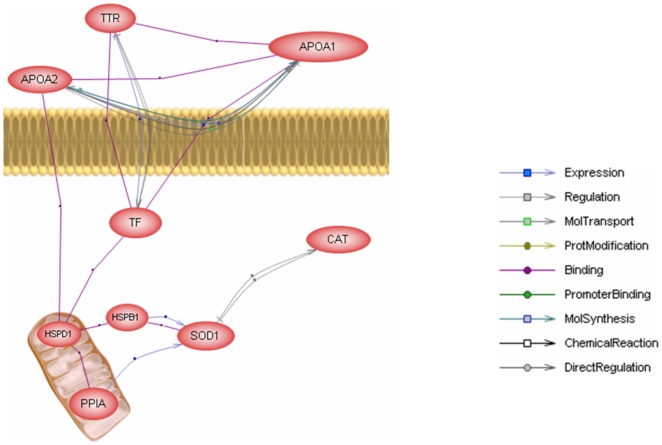
Proteins that were identified using MALDI-TOF MS analysis were examined using PathwayAssist. The pathway was built by looking for direct interactions of the down-regulated and up-regulated proteins. The predominant cluster from this analysis is depicted in this figure. Briefly, peptidyl-prolyl cis-trans isomerase A (PPIA) and heat shock protein 27 kda (HSPB1) increase the expression of superoxide dismutase-1 (SOD1), which regulates catalase (CAT). SOD1 expression is under negative regulation by CAT.

**Figure 8:**
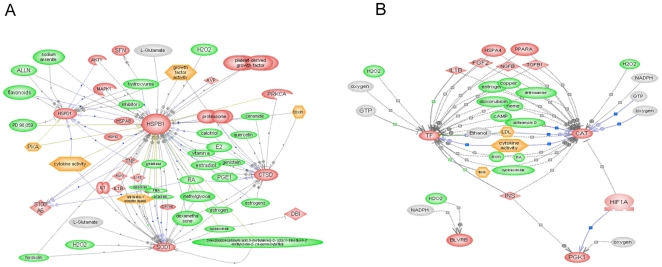
Common regulators for the up-regulated and down-regulated proteins were analyzed with the help of PathwayAssist. Heat shock protein 27 kda (HSPB1) and superoxide dismutase-1 (SOD1) are both central proteins in the pathway analysis performed with the up-regulated list (A). SOD1 is regulated by a number of cytokine molecules, and HSPB1 is directly associated with growth factors. Panel B shows the common regulators for the down-regulated proteins observed in this study. It is evident that CAT and crystal structures of mutant K206A, chain A (TF) is a key protein that interacts with a number of different signaling molecules in this pathway.

Proteins that were identified using MALDI-TOF MS analysis were examined using PathwayAssist. The pathway was built by looking for direct interactions of the down-regulated and up-regulated proteins. The predominant cluster from this analysis is depicted in Figure 7. Briefly, peptidyl-prolyl cis-trans isomerase A (PPIA) and heat shock protein 27 kda (HSPB1) increase the expression of superoxide dismutase-1 (SOD1), which regulates catalase (CAT). SOD1 expression is under negative regulation by CAT. In addition, common regulators for the up-regulated and down-regulated proteins were analyzed with the assistance of PathwayAssist. An examination of the up-regulated and down-regulated proteins in T1D patients with and without ESRD is shown in Figure 8. Heat shock protein 27 kda (HSPB1) and superoxide dismutase-1 (SOD-1) are both central proteins in the pathway analysis performed with the up-regulated list (Figure 8A). SOD1 is regulated by a number of cytokine molecules, and HSPB1 is directly associated with growth factors. Figure 8B shows the common regulators for the down-regulated proteins observed in this study. It is evident that CAT and crystal structures of mutant K206A, chain A (TF) is a key protein that interacts with a number of different signaling molecules in this pathway.

## Discussion

In this study we employed several different techniques such as proteomics, clinical biochemistry, electron microscopy, and immunohistochemistry to identify pathways of persistent cellular changes in skin biopsies of T1D patients. The effect of a KP transplant on cellular pathways, protein expression, and ultrastructural features was evaluated. We focused on the altered expression of several proteins involved in oxidative stress, aerobic and anaerobic glycolysis, and intracellular signaling normalized by KP transplant and combined them in molecular/ultrastructural studies.

T1D patients showed an up-regulation of HSP60, HSP27, MnSOD, and ATP synthase δ chain with a further increase in those patients with ESRD associated with T1D. This suggests that HSP and anti-oxidative machinery is entirely altered and thereafter restored by KP transplantation. These data are consistent with previous studies indicating that transient exposure of pancreatic islets to high glucose increases the activities of antioxidant enzyme, such as Cu/Zn-SOD [25]. HSP60 and HSP27 are synthesized in large amounts when cells are exposed to stressful stimuli such as inflammation, infection, and exposure to oxidizing agents [26], [27], [28], [29]. We also identified down-regulation of catalase, which has important antioxidant functions. Consequently, there is decreased ability to counteract increased oxidative stress in long-standing T1D. It has been reported that high levels of glucose can produce permanent chemical alterations in proteins, increase lipid peroxidation and production of free radicals in several experimental models of hyperglycemia [30], [31]. In addition to the above-mentioned group of proteins, proteomics data showed that long-standing T1D when associated with ESRD also regulates cytoplasmic proteins involved in aerobic and anaerobic glycolysis, gluconeogenesis, and mitochondrial electron transport. Interestingly, T1D+ESRD patients who were hyperglycemic and hyperinsulinemic also had increased triglycerides, which are produced by anaerobic glycolysis and up-regulated pyruvate kinase, while KP-transplanted patients were less dyslipidemic and presented lower pyruvate kinase levels than T1D+ESRD patients comparable with controls [20], [32]. T1D patients were analogous to T1D+ESRD patients as far as the expression of these three proteins, suggesting effect of T1D on these pathways. The last identified altered expressed proteins (stratifin, rotamase, S100 calcyclin) were involved in the intracellular signaling pathway. Lee and co-workers suggested [33] that rotamase may play a role in the folding of SOD-1 and in its dimerization, possibly explaining the up-regulation of rotamase in parallel to the up-regulation of SOD-1. In particular, this association was related to a calcium-dependent pro-apoptotic mechanism, and ultrastructural analysis emphasized the presence of apoptotic nuclei both in T1D patients and in T1D+ESRD patients.

Aiming to address if a parallel increase of redox state took place in the periphery, we evaluated malondialdehyde (MDA), a terminal compound derived from lipid peroxidation and from eicosanoid biosynthesis, widely used to monitor oxidative stress [25]. We measured both the free and the total MDA forms, the first being considered an index of recent damage and the second an index of prior damage. Therefore, we evaluated the levels of endogenous antioxidants such as GSH and GSSG. In T1D patients, increased MDA levels in plasma were evident consistently with the increased oxidative status than the controls, while KP-transplanted patients presented lower MDA levels and were comparable to the controls. We did not observe any differences in GSH levels, which counteract the effect of free radicals. This is consistent with a previous study from our group, which showed that KP transplantation can reduce the levels of MDA [34].

The ultrastructural alterations found in T1D and T1D+ESRD skin biopsies included thickening of the capillary basal membrane, collapse of vessel lumen, and microvillar ramification. We observed that basal membranes were thicker in T1D and T1D+ESRD groups compared with controls, and that these alterations were corrected in patients who had a KP transplant for at least five years. We note that basal membrane thickening was particularly evident in T1D+ESRD patients. The lumen of the vessels was collapsed and microvilli were more branched in the same group. Moreover, the T1D+ESRD group had an apoptotic pattern of endothelial cells consistent with previous studies that describe a role of hyperglycemia in inducing apoptosis in endothelial cells [35]. All of these alterations were somehow more evident in T1D+ESRD compared with T1D patients, possibly due to the coexistence of two “toxic” situations, i.e., uremia and hyperglycemia that may act additively. Skin biopsies from KP-transplanted patients presented an impressive improvement of ultrastructural alterations (basal membrane thickening, collapse of vessel lumen, microvillar ramifications), as previously described in kidney-transplanted patients who received islet transplantation [36]. It is well known that hyperglycemia and diabetes induce oxidative stress responses in animal models and cell culture systems [37], [38], [39]. However, few studies have employed human tissues to study the biochemistry of diabetic complications [33], [39], [40], [41]. The improvement of ultrastructural abnormalities is consistent with what has been reported in the literature by Eberl and co-workers, who showed that long-term blood glucose normalization achieved by pancreas transplantation improved most skin microcirculation parameters with a positive effect on functionality of the skin [42].

These findings are consistent with the hypothesis that hyperglycemia and uremia, through different mechanisms, determine persistent cellular changes of the oxidative status and pathways and that restoration of normoglycemia with KP transplantation can correct most of these biochemical abnormalities. To a lesser extent, T1D not associated with ESRD is also characterized by an increase of oxidative stress. It is not clear if the alterations of these pathways may determine alteration at chromatin levels and altered DNA repairing. The next logical step will be to evaluate the status of DNA damage during the normalization of these pathways after kidney-pancreas transplantation.

Some of these proteins or pathways addressed in our study may become either biomarkers of oxidative stress *in vivo* or could be potential therapeutic targets of a new class of drugs aimed at correcting persistent cellular changes when normoglycemia cannot be restored.

## Supporting Information

File S1 Online Supplementary Materials and Methods.
(0.06 MB DOC)
