# Supplementary material for: Proteomics Reveals Novel Oxidative and Glycolytic Mechanisms in Type 1 Diabetic Patients' Skin Which Are Normalized by Kidney-Pancreas Transplantation
Source: PLoS One. 2010 Mar 29;5(3):e9923. doi: 10.1371/journal.pone.0009923 (PMC2848014; doi:10.1371/journal.pone.0009923)
Supplement: File S1 — Online Supplementary Materials and Methods. (0.06 MB DOC) [file pone.0009923.s001.doc]

#### *ONLINE SUPPLEMENTAL MATERIAL*

#### *Materials*

Paraformaldehyde, glutaraldehyde, cacodylate buffer, and osmium tetroxide were from Merck (Darmstadt, Germany), ethanol from Carlo Erba Reagenti (Milan, Italy), Epon-Araldite from FLUKA (Switzerland), 200-mesh nickel grids and uranyl acetate from Polysciences Inc. (Warrington, PA), Formvar film, Reynold’s lead citrate from Sigma Chemical Co. (St. Louis, MO), bovine trypsin from (Roche, Basel, Switzerland); μZipTipC_18_ from Millipore Corp. (Bedford, MA), 2D IEF/SDS–PAGE Immobiline IPG strips DryStrips (18 cm 3–10 pH non-linear) and IPGphor apparatus from Amersham Biosciences (Piscataway, NJ), and Bio-Rad for protein assay (Hercules, CA).

***Antibodies***

The following antibodies and antisera were used to characterize the skin biopsy specimens: anti-HSP60 (Chemicon International, Temecula, CA; monoclonal at 1:100 dilution), anti-HSP27 (Novocastra Laboratories Ltd, Newcastle upon Tyne, UK; monoclonal at 1:40 dilution), anti-S100 (Dako, Copenhagen, DK; polyclonal at 1:100 dilution), anti-ACBP (offered by Dr. Nils Faergeman of Syddansk Universitet, Odense; polyclonal at 1:2000 dilution), anti-cathepsin D (Santa Cruz Biotechnology, Inc., Santa Cruz, CA; polyclonal at 1:100 dilution), anti-PRX V (Santa Cruz Biotechnology, Inc.; polyclonal at 1:50 dilution), and anti-Catalase (Novus Biologicals, Inc., Littleton, CO; polyclonal at 1:100 dilution).

***Instruments***

We used the following instruments: Philips Morgagni 268D electron microscope (FEI Co., Netherlands); Image Scanner and Image Master 2D Elite software (Amersham Biosciences, Uppsala, Sweden); ProXCISION for automated excision (PerkinElmer, Waltham, MA); and MALDI-TOF MS analysis (Voyager-DE STR from Applied Biosystem, Framingham, MA). For GC–MS analysis we used: Hewlett–Packard 5890 gas chromatograph (Hewlett–Packard Co., Palo Alto, CA) coupled to a 5988A mass spectrometer equipped with an HP-5 fused silica capillary column (25 m, 0.32 mm i.d., 0.25 mm film thickness).

#### *Pathway Analysis*

Analysis of the down-regulated and up-regulated protein data identified by MALDI-TOF MS analysis was performed using PathwayAssist (Ariadne Genomics, Rockville, MD). Protein lists were imported into PathwayAssist for visualization of biological pathways identified using the ResNet™ database that contains over 500,000 functional relationships.

***Immunohistochemistry***

For immunohistochemical stains, skin tissues were formalin-fixed and routinely paraffin embedded. Five μm-thick sections were mounted on poly-L-lysine coated slides, deparaffinized, and hydrated through graded alcohol to water. Endogenous peroxidase activity was inhibited by dipping sections in 3% hydrogen peroxide for 10 minutes at room temperature. After heat antigen retrieval, performed using microwave oven or enzymatic treatments, primary antibody incubation was performed at 4 °C for 18-20 hours and followed by the avidin-biotin complex (ABC) procedure [1]. Sections were then immersed in 0.03% 3,3’ diaminobenzidine tetrahydrochloride and counterstained with Harris’s hematoxylin.

***Electron microscopy***

For ultrastructural studies, skin-punch biopsies were fixed for 2 hours at 4°C in a mixture of 2% paraformaldehyde and 2% glutaraldehyde in 0.05 M cacodylate buffer, pH 7.3. Samples were post-fixed in 1% osmium tetroxide for 1 hour at room temperature, then dehydrated and embedded in Epon-Araldite. Ultrathin sections were cut with a diamond knife and mounted on 200-mesh nickel grids, previously coated with a Formvar film. Ultrathin Epon-embedded sections were stained with aqueous uranyl acetate and Reynold’s lead citrate solutions and subsequently examined with a Philips Morgagni 268D electron microscope. To evaluate the basal membrane of vessels, the collapse of vessel lumens, the microvillar ramification, the dilatation of the endoplasmic reticulum, the presence of intermediate filaments and Weibel Palade granules and the nuclear aspects, 15 vessels were examined for each case, as previously described [2]. The measurement of the basal membrane and the collapse of the lumen were performed using the “Measure arbitrary distance” and the “Measure arbitrary area” tool of AnalySIS Image Processing 3.0 software (Soft Imaging System GmbH, Münster, Germany), which allowed us to measure the thickness of the basal membrane and the area of vessel lumen.

***Malondialdehyde and GSH quantification***

Blood samples were collected in vacutainer tubes containing EDTA as an anticoagulant and centrifuged at 4°C at 3,000 rpm for 10 minutes. The plasma was removed and immediately analyzed or stored at –80°C. Free and total (free+bound) malondialdehyde (MDA) levels were evaluated by a “reference method” based on gas chromatography-mass spectrometry technique with isotope dilution (ID-GC-MS). GC-MS conditions, sample preparation, MDA evaluation, and calculation were performed as previously described [3,4]. Total sulfhydryl groups were determined in plasma samples using DTNB (Elmann’s reagent, 5,5-Dithio-bis-2-nitrobenzoic acid). Bound and sulfhydryl groups were measured after separation of proteic component with TCA precipitation method according to published procedures [5].

**References**

1. Hsu SM, Raine L (1981) Protein A, avidin, and biotin in immunohistochemistry. J Histochem Cytochem 29: 1349-1353.

2. Fiorina P, Folli F, Maffi P, Placidi C, Venturini M, et al. (2003) Islet transplantation improves vascular diabetic complications in patients with diabetes who underwent kidney transplantation: a comparison between kidney-pancreas and kidney-alone transplantation. Transplantation 75: 1296-1301.

3. Shevchenko A, Wilm M, Vorm O, Mann M (1996) Mass spectrometric sequencing of proteins silver-stained polyacrylamide gels. Anal Chem 68: 850-858.

4. Cighetti G, Allevi P, Anastasia L, Bortone L, Paroni R (2002) Use of methyl malondialdehyde as an internal standard for malondialdehyde detection: validation by isotope-dilution gas chromatography-mass spectrometry. Clin Chem 48: 2266-2269.

5. Cighetti G, Debiasi S, Paroni R, Allevi P (1999) Free and total malondialdehyde assessment in biological matrices by gas chromatography-mass spectrometry: what is needed for an accurate detection. Anal Biochem 266: 222-229.
